# From Differential DNA Methylation in COPD to Mitochondria: Regulation of AHRR Expression Affects Airway Epithelial Response to Cigarette Smoke

**DOI:** 10.3390/cells11213423

**Published:** 2022-10-29

**Authors:** Qing Chen, Kingsley Okechukwu Nwozor, Maarten van den Berge, Dirk-Jan Slebos, Alen Faiz, Marnix R. Jonker, H. Marike Boezen, Irene H. Heijink, Maaike de Vries

**Affiliations:** 1 University of Groningen, University Medical Center Groningen, Department of Pathology and Medical Biology, 9713 GZ Groningen, The Netherlands; 2 University of Groningen, University Medical Center Groningen, Groningen Research Institute for Asthma and COPD (GRIAC), 9713 GZ Groningen, The Netherlands; 3 Centre for Heart Lung Innovation, Department of Anesthesiology, Pharmacology & Therapeutics, The University of British Columbia, Vancouver, BC V6Z 1Y6, Canada; 4 University of Groningen, University Medical Center Groningen, Department of Pulmonology Disease, 9713 GZ Groningen, The Netherlands; 5 Respiratory Bioinformatics and Molecular Biology (RBMB), School of Life Sciences, University of Technology Sydney, Sydney, NSW 2007, Australia; 6 University of Groningen, University Medical Center Groningen, Department of Epidemiology, 9713 GZ Groningen, The Netherlands

**Keywords:** COPD, cigarette smoking, AHRR, airway epithelial cells, CRISPR/Cas9, proliferation, mitochondrial integrity, cell death modalities

## Abstract

Cigarette smoking causes hypomethylation of the gene Aryl Hydrocarbon Receptor Repressor (*AHRR*), which regulates detoxification and oxidative stress-responses. We investigated whether *AHRR* DNA methylation is related to chronic obstructive pulmonary disease (COPD) and studied its function in airway epithelial cells (AECs). The association with COPD was assessed in blood from never and current smokers with/without COPD, and in AECs from ex-smoking non-COPD controls and GOLD stage II-IV COPD patients cultured with/without cigarette smoke extract (CSE). The effect of CRISPR/Cas9-induced AHRR knockout on proliferation, CSE-induced mitochondrial membrane potential and apoptosis/necrosis in human bronchial epithelial 16HBE cells was studied. In blood, DNA methylation of *AHRR* at cg05575921 and cg21161138 was lower in smoking COPD subjects than smoking controls. In vitro, *AHRR* DNA methylation at these CpG-sites was lower in COPD-derived than control-derived AECs only upon CSE exposure. Upon *AHRR* knockout, we found a lower proliferation rate at baseline, stronger CSE-induced decrease in mitochondrial membrane potential, and higher CSE-induced late apoptosis/necroptosis. Together, our results show lower DNA methylation of *AHRR* upon smoking in COPD patients compared to non-COPD controls. Our data suggest that higher airway epithelial *AHRR* expression may lead to impaired cigarette smoke-induced mitochondrial dysfunction and apoptosis/necroptosis, potentially promoting unprogrammed/immunogenic cell death.

## 1. Introduction

Chronic obstructive pulmonary disease (COPD) is characterized by persistent airflow limitation and reduced gas exchange due to abnormalities of the airways and alveoli [1]. These abnormalities are caused by inflammation, damage and abnormal tissue repair, leading to mucus hypersecretion (chronic bronchitis), small airway wall thickening and/or destruction of the alveoli (emphysema). The main symptoms of COPD are cough, shortness of breath and expectoration of sputum, which severely influence the life quality and life span of patients suffering from COPD [2]. COPD is among the top three causes of death worldwide, and its burden is expected to increase in the upcoming decades with the increasing age of the population [1].

The main risk factor for COPD is the inhalation of noxious particles, such as cigarette smoke, air pollutants and job-related exposures [1]. When inhaled, cigarette smoke first encounters the airway epithelium, which forms the first barrier to protect sub-epithelial layers from inhaled insults such as cigarette smoke, and is involved in the initiation of pro-inflammatory activity after exposure to cigarette smoke [3]. In the airway epithelium, cigarette smoke inflicts damage to the mitochondria [4,5]. This leads to the production of reactive oxygen species (ROS), which in turn cause cellular damage and activation of pro-inflammatory responses [6]. Furthermore, cigarette smoke has been shown to induce a switch from apoptotic to necrotic cell death, resulting in the release of damage-associated molecular patterns (DAMPs), including double-stranded DNA (dsDNA) [7,8]. These DAMPs play a detrimental role in the development of COPD, causing neutrophilic inflammation and dysregulation of lung tissue repair processes [9]. To detoxify inhaled cigarette smoke, the xenobiotic metabolism is activated in epithelial cells upon smoking, which is protective on one hand, but on the other hand can further increase the generation of ROS and mitochondrial damage in a vicious cycle [10,11]. These processes are thought to contribute to the pathogenesis of COPD. However, it is still unclear why there are individual differences in the susceptibility of the airway epithelium to cigarette smoke.

Not all smokers develop COPD and differences in (epi)genetics are thought to contribute to the variability in the susceptibility for COPD. Many environmental exposures, including inhaled cigarette smoke, are known to alter gene expression through epigenetic modifications. DNA methylation is an epigenetic mechanism involving the transfer of a methyl group to a cytosine nucleotide adjacent to a guanine nucleotide, a so-called CpG-site. DNA methylation often occurs in the regulatory regions of the DNA, leading to a decrease in gene expression [12,13]. Of interest, DNA methylation has been shown to be highly associated with cigarette smoking. For instance, large-scale epigenome-wide association studies (EWAS) consistently reported hypomethylation of the aryl hydrocarbon receptor repressor (AHRR) to be associated with cigarette smoking as well as lower lung function [14,15]. Moreover, lower DNA methylation at the CpG-sites cg05575921 and cg21161138 in the *AHRR* gene in human lung tissue was not only associated with smoking, but also associated with higher *AHRR* mRNA expression [13]. Studies on the functional role of *AHRR* primarily focused on its repressive effect on the expression of the aryl hydrocarbon receptor (*AHR*). *AHR* is activated by aryl hydrocarbon ligands in cigarette smoke, such as dioxin. Once activated, AHR translocates to the nucleus to induce the transcription of genes related to xenobiotic metabolism, cell cycle arrest and mitochondria-related apoptosis, including expression of *CYP1A1*, *BAX* and *P27* [16]. Similar to cell cycle inhibitor *P27*, *P21* expression was found to be increased by AHR activator compound TCDD or *AHR* mRNA overexpression [17,18]. Thus, AHR is thought to have a protective effect upon cigarette smoking, also with respect to the development of lung cancer [19,20]. Another downstream target gene of *AHR* is its repressor *AHRR*, thereby forming a negative feedback loop [21]. Whether cigarette smoke differently affects DNA methylation of *AHRR* in the airway epithelium of COPD patients compared to smokers who do not develop COPD, and whether it contributes to pathological processes in the lungs is currently unknown.

We hypothesized that epithelial cells from COPD patients are more susceptible to cigarette smoke-induced hypomethylation of *AHRR*. Subsequent higher expression of *AHRR* may alter epithelial responses to cigarette smoke, including mitochondria-related apoptosis. Therefore, in this study, we first investigated the association between COPD and DNA methylation of *AHRR* at cg05575921 and cg21161138 in whole blood from never and current smokers with and without COPD and in airway epithelial cells from COPD patients and non-COPD controls. Secondly, we investigated the functional role of AHRR in bronchial epithelial cell line 16HBE by taking advantage of CRISPR-Cas9 knockout of the gene. 

## 2. Materials and Methods

### 2.1. Characteristics of the Subjects Included in the Study

#### 2.1.1. Subject Characteristics of the Lifelines Cohort Study

DNA methylation data was available for 1561 subjects who were non-randomly selected from the Lifelines general population-based cohort study based on smoking history (never or current smoker with >5 pack years), with and without COPD as defined by pre-bronchodilator FEV1/FVC< 70% and job-related exposures [22,23]. Written informed consent was provided by all included subjects and the study was approved by the Medical Ethics Committee of the University Medical Center Groningen (METc 2007/152). Subject characteristics are shown in Table 1. 

#### 2.1.2. Subject Characteristics of the Donors of the Primary Airway Epithelial Cells

Airway epithelial cells (AECs) were obtained from 14 ex-smoking COPD patients with GOLD stage II/III/IV and 8 age- and sex-matched ex-smoking controls with normal lung function. Ex-smokers were defined as subjects who had a smoking history of at least 5 pack years and stopped smoking for at least 6 months. All donors gave written informed consent and ethical approval was given by the Medical Ethics Committee of the University Medical Center Groningen (METc 2014/102 and METc 2016/572). The subject characteristics of the donors are shown in Table 2.

### 2.2. DNA Methylation Measurement in the Lifelines Cohort

DNA methylation levels were determined in whole blood using the Illumina Infinium Human Methylation 450K Array and presented as beta values. We focused on the CpG-sites cg05575921 and cg21161138 in the *AHRR* gene.

### 2.3. Cell Culture

#### 2.3.1. Isolation and Culture of Primary AECs from COPD Patients and Non-COPD Controls

AECs were isolated from bronchial brushes, stored in liquid nitrogen and cultured as described previously [24]. In short, AECs were seeded into T25 culture flasks coated with 30 μg/mL collagen (CellSystem, Troisdorf, Germany), 30 μg/mL fibronectin (Sigma-Aldrich, St. Louis, MO, USA) and 10 μg/mL BSA (Sigma-Aldrich) in hormonally supplemented Airway Epithelium Cell Basal Medium (AEBM; PromoCell, Heidelberg, Germany) with 100 U/mL Penicillin and 100 mg/mL streptomycin (Gibco, Waltham, MA, USA), cultured at 37 °C with 5% CO_2_ and used for experiments at passage 3.

#### 2.3.2. Culture of 16HBE Cells

The human bronchial epithelial cell line 16HBE was generously offered by D.C. Gruenert (University of California, San Francisco, CA, USA). 16HBE cells were cultured in EMEM (LONZA, Walkersville, MD, USA) with 2 mM GlutaMax (Life Technologies, Paisley, UK), 100 U/mL Penicillin and 100 mg/mL streptomycin, and 10% fetal calf serum (FCS; Sigma-Aldrich) in T25 flasks coated with 30 μg/mL collagen and 10 μg/mL BSA at 37 °C with 5% CO_2_. When 90% confluent, the cells were passaged or seeded in duplicates into coated 24-wells plates for experiments. 

### 2.4. Generation, Selection and Culture of Wild-Type and AHRR Knockout 16HBE Cells 

The CRISPR-Cas9 plasmid Px458 was used to generate AHRR knockout (KO) cell lines cells as previously described [25,26]. Guide RNA (gRNA) was designed using the online tool Benchling and generated by Sigma-Aldrich. Plasmids were cloned in NEB 5-alpha competent Escherichia coli (New England Biolabs, Frankfurt am Main, Germany), selected using ampicillin positive (100 µg/mL) plates, extracted using the QIAprep Spin Miniprep Kit (Qiagen, Valencia, CA, USA). Cells were transfected in 3 µL of Lipofectamine 3000 (Invitrogen, Carlsbad, CA, USA) diluted in 62.5 µL of Opti-MEM (ThermoFisher Scientific, Waltham, MA, USA) and combined with a DNA mixture containing 1250 ng of purified plasmid, 4 µL of P3000 reagent (Invitrogen, Carlsbad, CA, USA) and 125 µL of Opti-MEM (Thermofisher Scientific) for 4 h at 37 °C with 5% CO_2_. Wild type 16HBE (WT) cells were taken along the procedure without genetic editing as proper controls of the knockout cell lines. Single clones were selected by FACS gating for green fluorescent protein positive cells, cultured as described for 16HBE cells and expanded separately until sufficient cells to be cultured in T25 culture flasks. Genomic DNA was isolated, amplified using PCR and the PCR product was Sanger sequenced to confirm the knockout of AHRR (Baseclear, Leiden, The Netherlands). Sanger sequencing results were analyzed using the online tools Benchling and Tracking of Indels by Decomposition: TIDE.

### 2.5. Preparation of Cigarette Smoke Extract (CSE)

Kentucky 3R4F research-reference cigarettes (The Tobacco Research Institute, University of Kentucky, Lexington, KY, USA) were used to prepare CSE. Filters were removed and the cigarettes were smoked using a hose pump (Watson Marlow 603S, Cornwall, UK) at a rate of 7 L/h [8]. The smoke of two cigarettes was bubbled through 25 mL EMEM medium (for 16HBE cells) or AEBM medium (for AECs) and defined as 100% CSE. The extract was used freshly within 15 min after preparation.

### 2.6. In Vitro Cell Treatment

#### 2.6.1. Treatment of AECs

When 90% confluent in T25 flasks, AECs were seeded into 24-wells plates in duplicates at a density of 50,000 cells/well, grown for 2–3 days until 90% confluence was reached and hormonally deprived overnight. Next, cells were incubated with or without 7.5% CSE for 24 h, harvested with TRIzol for RNA and DNA isolation according to the manufacturer’s guidelines (Sigma-Aldrich).

#### 2.6.2. Treatment of 16HBE Cells

To assess mRNA expression of *AHRR*, 16HBE cells were seeded in duplicate at a density of 50,000/well in collagen-coated 24-well plates. After 3 days at ~90% confluence, cells were serum-deprived and stimulated with 10% CSE, 20% CSE or EMEM control for 6 h before harvesting for RNA isolation and qPCR.

For the cell proliferation assay, WT and AHRR KO 16HBE cells were seeded into collagen-coated 96-wells plates at a density of 2000 cells/well in 200 µL/well and the MTS assay was performed at day 2, 4 and 6 as described below. 

For the other assays, WT cells were seeded at a density of 40,000 cells/well and AHRR KO cells at a density of 55,000 cells/well to adjust for differences in proliferation rate. At ~90% confluence, cells were serum-deprived, incubated with/without 10 or 20% CSE for 4, 6 or 24 h and harvested for isolation of RNA, DNA, flow cytometry or collection of cell-free supernatant.

### 2.7. RNA and DNA Isolation

Cells were harvested using TRIzol (MRC, Cincinnati, OH, USA) solution and total RNA was isolated following the user guide of TRIzol solution and quantified using a Nanodrop-1000 (ND 2.0; NanoDrop Technologies, Wilmington, DE, USA). One µg of total RNA from each sample was reverse-transcribed into cDNA using the iScript cDNA synthesis kit (Bio-Rad Laboratories, Utrecht, The Netherlands) according to the manufacturer’s protocol. For DNA methylation analysis, the DNA remaining in the phenol-chloroform phase was precipitated with pure ethanol and centrifuged at 2000× *g* to pellet the DNA. The pellet was washed twice with 0.1 M sodium citrate in 10% ethanol and subsequently washed twice with 75% ethanol. DNA pellets were air dried on ice and solubilized with 8 mM NaOH and stored at −20 °C. The yield was quantified using Nanodrop-1000 as described above. 

### 2.8. DNA Methylation Analysis

DNA was bisulfite converted using the EZ DNA methylation Gold Kit (Zymo Research, Freiburg im Breisgau, Germany) according to manufacturer’s instructions. Primers were developed with the Pyromark Assay Design 2.0 (Qiagen, Hilden, Germany) and complete primers sequences are listed in Table 3 below. Fifty nanograms of the bisulfite converted DNA was used as template for each of the subsequent PCRs. DNA was amplified for 45 cycles with the Applied Biosystems Veriti Thermal Cycler (ThermoFisher Scientific) using the HotStarTaq Mastermix (Qiagen). DNA quality was checked using DNA agarose gel electrophoresis before CpG methylation was quantified using the Pyromark Q24 Autoprep System (Qiagen) with the PyroMark Q24 Advanced CpG Reagents (Qiagen). Methylation values were determined with the Pyromark Q24 Software (Qiagen) and expressed as percentage. Quality of the pyrosequencing was assessed manually for both CpG-site in all subjects individually. In brief, samples were excluded when the pyrogram met the following criteria: more than one identified peak at one base of the CpG-site; large variation compared to the predicted curve; insufficient DNA input according to DNA agarose gel electrophoresis (peaks below an intensity of 100).

### 2.9. qPCR

qPCR was performed in duplicates using GoTaq(R) Probe qPCR Master Mix kits (Promega Benelux, Leiden, The Netherlands) on a QuantStudio ViiA7 Real-Time PCR System (ThermoFisher Scientific), and the result was analyzed using QuantStudio Software v1.6.1 (ThermoFisher Scientific). *B2M* and *PPIA* were used as housekeeping genes. Taqman probes (see Table 4) were purchased from ThermoFisher Scientific.

### 2.10. Cell Proliferation Assay

The proliferation rate was assessed using CellTiter 96^®^ AQueous Non-Radioactive Cell Proliferation Assay (Promega, Leiden, The Netherlands), assessing metabolic activity as read-out for cell number. The assay was performed on day 2, day 4 and day 6 after seeding. [3-(4,5-dimethylthiazol-2-yl)-5-(3-carboxymethoxyphenyl)-2-(4-sulfophenyl)-2H-tetrazolium] (MTS) and phenazine methosulfate solution (PMS) was mixed into a 1:20 ratio before use and 20 µL of the mixture was added to the wells of a 96-wells plate, incubated for 1.5 h at 37 °C with 5% CO_2_. In the presence of dehydrogenase enzymes in metabolically active cells, MTS is converted into soluble state. The reaction was stopped by adding 25 μL/well 10% SDS and the quantity of formazan was measured by the amount of 490 nm absorbance using Ultra Microplate Reader (EL808; Bio-Tek Instruments, Bad Friedrichshall, Germany). The readout is directly proportional to the number of living cells in culture.

### 2.11. Mitochondrial Membrane Potential (Δψm) Assay 

Mitochondrial membrane potential was assessed using the fluorescent dye tetramethylrhodamine ethyl ester (TMRE; Abcam, Cambridge, UK). Cells were washed with PBS with 0.9 mM CaCl_2_ and 0.49 mM MgCl_2_ before labeling with TMRE at 37 °C for 15 min. Treatment with 50 µM Carbonyl cyanide 3-chlorophenylhydrazone (CCCP, Sigma-Aldrich) for 10 min was used as the positive control. After TMRE incubation, the fluorescent signal was measured in the PE channel using BD FACSVerse (BD Biosciences, San Jose, CA, USA).

### 2.12. Live/Dead Cell Analysis

Cells were washed with Cell Staining Buffer (BioLegend, Amsterdam, The Netherlands) and labeled for annexin V–(FITC)/propidium iodide (PI) using Annexin V Binding Buffer (BioLegend) according to the manufacturer’s instructions (IQproducts, Groningen, The Netherlands) and analyzed using BD FACSVerse flow cytometer.

### 2.13. Cytochrome C ELISA Assay

Cytochrome C levels in cell-free supernatants were measured using a Human Cytochrome C Quantikine ELISA Kit (R&D systems, Abingdon, UK) according to the manufacturer’s instructions. 

### 2.14. dsDNA Measurement Assay

Release of dsDNA in cell-free supernatants was analyzed using the Quant-iTPicogreen assay (Invitrogen, Breda, The Netherlands) and a FL600 fluorescence plate reader (Bio-Tek Instruments) with a wavelength of 485 nm through a 590 nm band pass filter.

### 2.15. Statistics

#### 2.15.1. DNA Methylation Data Analysis in Lifelines

To test if DNA methylation levels at cg05575921 and cg21161138 were associated with COPD, we performed robust logistic regression analyses in R with COPD as outcome and DNA methylation as predictor separately for never smokers and current smokers. The analyses were adjusted for the potential confounders age, sex, pack-years (only in the current smokers), batch effects and white blood cell composition. 

#### 2.15.2. In Vitro Experiments

For differences between groups (COPD vs. non-COPD controls or AHRR KO vs. WT 16HBE cells) groups, we used the Mann–Whitney test. For paired measurements within the groups upon exposure to CSE, we used the Wilcoxon signed-rank test. *p* < 0.05 was considered significant.

## 3. Results

### 3.1. DNA Methylation Levels of AHRR Are Lower in Subjects with COPD Compared to Non-COPD Controls in Current Smokers

**Figure 1:**
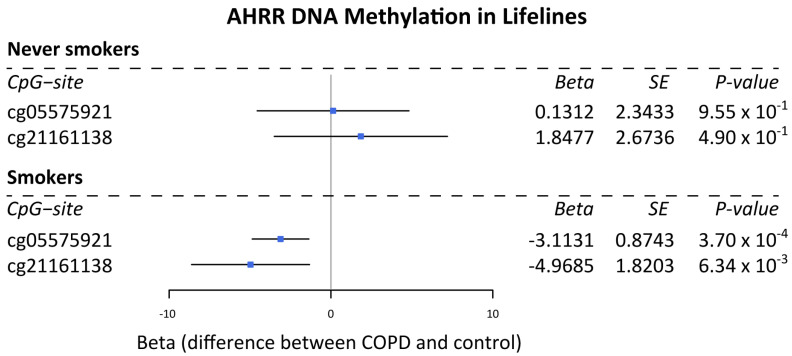
Association of AHRR DNA methylation with COPD in never smokers and smokers from Lifelines. Forest plot of the association between COPD (defined as FEV1/FVC < 70%) and DNA methylation at CpG-sites cg05575921 and cg21161138 in the gene AHRR in whole blood from 658 current smokers and 903 never smokers from the Lifelines DNA methylation cohort study. Beta indicates the effect estimate, i.e., the difference between COPD and control subjects. A negative value points towards a negative association.

DNA methylation levels at the two *AHRR* CpG-sites cg05575921 and cg21161138 have been previously described to be reduced upon smoking and lower in smokers versus never smokers [13]. Here, we studied if DNA methylation at these CpG-sites is additionally associated with COPD, which we assessed in never smokers and current smokers with and without COPD separately. We found that DNA methylation levels at both cg05575921 (*p* = 0.00037) and cg21161138 (*p* = 0.00634) were lower in the blood of COPD patients compared to non-COPD controls in current smokers only (Figure 1). In never smokers, no difference was observed between subjects with a normal lung function and those with (mild) COPD—likely due to other environmental exposures such as job-related exposures. 

### 3.2. Lower AHRR DNA Methylation in COPD- vs. Non-COPD-Derived AECs upon CSE Exposure 

**Figure 2:**
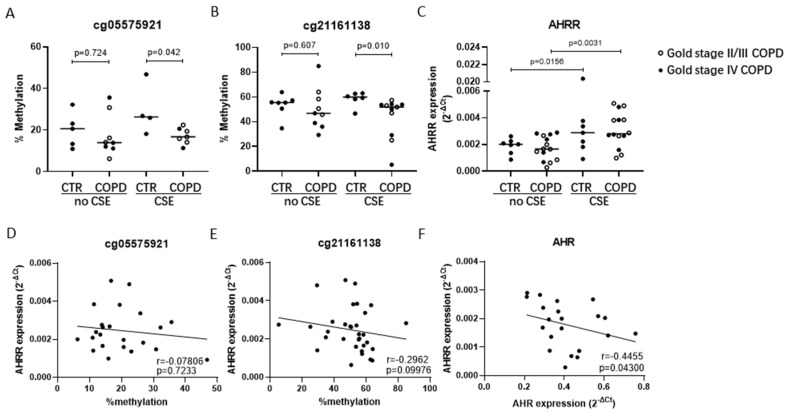
DNA methylation levels and mRNA expression of *AHRR* and the correlation of *AHRR* mRNA levels with *AHRR* DNA methylation or *AHR* expression in airway epithelial cells (AECs). AECs from 8 ex-smoking non-COPD controls (CTR) and 14 COPD (GOLD stage II-IV) patients were cultured until confluence, hormonally deprived overnight and incubated in medium with/without 7.5% CSE for 24 h. DNA and RNA were isolated for assessment of DNA methylation and mRNA expression levels. DNA methylation was assessed at CpG-sites cg05575921 (**A**) and cg21161138 (**B**) of *AHRR* by pyrosequencing. Samples that did not pass the pyrosequencing quality control were excluded. *AHRR* mRNA expression was assessed by qPCR (**C**), related to the expression of housekeeping genes *B2M* and *PPIA* and expressed as 2^−^^ΔCt^. Correlation between *AHRR* mRNA expression and DNA methylation levels of cg05575921 (**D**) or cg21161138 (**E**) in AECs exposed to 7.5% CSE or medium control (no CSE) from non-COPD controls and COPD patients. The correlation of mRNA expression between *AHRR* and *AHR* (**F**) in non-exposed AECs from non-COPD controls and COPD patients. Differences between CTR and COPD were tested using the Mann–Whitney test, differences between no CSE and CSE were tested using the Wilcoxon rank-sum test and correlations were tested using Spearman correlation test. *p*-values and median levels are indicated. In COPD groups, the empty symbols indicate GOLD stage II and III patients, and the filled symbols indicate GOLD stage IV COPD patients.

Next, we tested whether AHRR DNA methylation at CpG-sites cg05575921 and cg21161138 was also different in airway epithelium from subjects with and without COPD, incubated with/without 7.5% CSE for 24 h. In each of the subject and treatment groups, we had several drop-outs due to insufficient quantity and/or quality of the DNA, as reflected by the different number of dots in Figure 2. In line with our findings in blood, DNA methylation levels at both CpG-sites were significantly lower in AECs from COPD patients compared to non-COPD controls, but only upon exposure to CSE. No differences between COPD and non-COPD controls were observed in absence of CSE. CSE exposure itself did not have a significant effect on DNA methylation (online Supplemental Figure S1). No differences in DNA methylation at any of the CpG-sites were observed between mild-moderate (GOLD stage II-III) and severe (GOLD stage IV) COPD patients (Figure 2A,B). Exposure to CSE significantly induced the mRNA expression of AHRR in AECs derived from both COPD patients and non-COPD controls (Figure 2C). For cg21161138, but not for cg05575921, we observed a trend for a negative correlation between DNA methylation levels and mRNA expression of AHRR (Figure 2D,E). As expected, we found a significant negative correlation between the expression levels of AHR and AHRR, which was only assessed at baseline as cigarette smoke also directly regulates AHR expression independent of AHRR [27] (Figure 2F).

### 3.3. AHRR Knockout in 16HBE Cells

**Figure 3:**
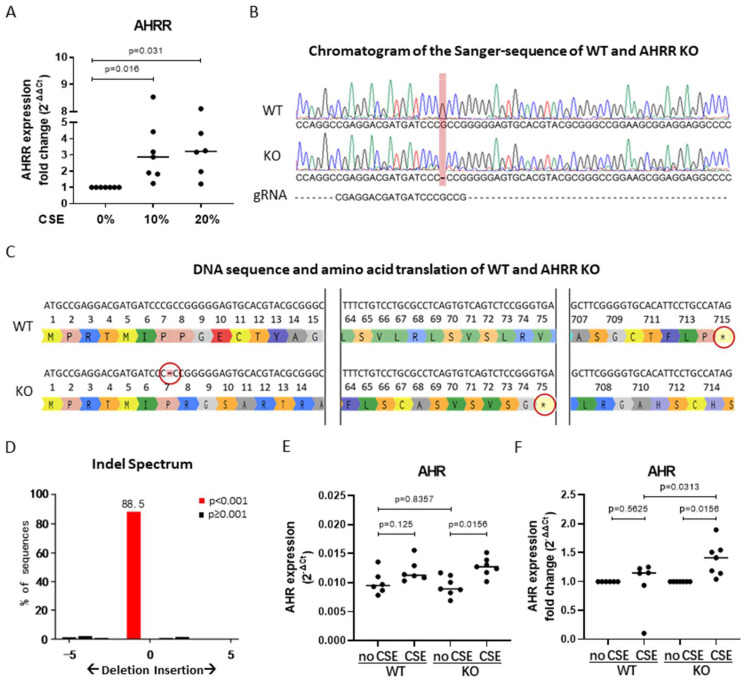
Induction of AHRR mRNA expression in 16HBE cells upon CSE exposure and validation of the AHRR CRISPR/Cas9 knockout. 16HBE cells (*n* = 7) were seeded in duplicate, grown to confluence, serum deprived overnight and exposed to 10% and 20% CSE or medium control for 6 h. Cells were harvested for RNA isolation. mRNA Expression of *AHRR* was measured by qPCR, related to the expression of housekeeping genes *B2M* and *PPIA* and expressed as 2^−^^ΔΔCt^ (**A**). AHRR knockout (KO) cells were generated using CRISPR/Cas9 together with 16HBE wild-type (WT) cells and assessed by Sanger sequencing. The base pair deletion is visualized by the chromatograms of the Sanger-sequenced 16HBE WT and AHRR KO cells as well as the sequence of the guide RNA (gRNA) (**B**). DNA sequence and amino acid translation of WT and AHRR KO in which the base pair deletion in the AHRR KO is indicated by a hyphen and a red circle. Stop codons are indicated by an asterisk and highlighted by red circles (**C**). Indel spectrum produced by Tracking of Indels by Decomposition (TIDE) analysis shows the base pair deletion (**D**). 16HBE WT or AHRR KO cells (*n* = 6/group) were seeded in duplicate, serum deprived overnight and exposed to 10% and 20% CSE or medium control (no CSE) for 6 h. Cells were harvested for RNA isolation *AHR* mRNA expression was measured by qPCR, related to the expression of housekeeping genes *B2M* and *PPIA* and expressed as 2^−^^ΔΔCt^ (**E**) or fold change (2^−^^ΔΔCt^) with the CSE groups normalized to their medium controls (**F**). Median levels are indicated. Differences between treatment conditions were tested with the Wilcoxon rank-sum test and the difference between WT and AHRR KO at baseline was tested with Mann–Whitney test. *p*-values are as indicated between the groups.

To further unravel the role of AHRR in airway epithelial responses in COPD, we used wild-type (WT) and AHRR knockout (KO) 16HBE cells as a model. As shown in Figure 3A, similar to the effect observed in AECs, AHRR mRNA expression was significantly increased upon exposure to CSE, confirming the suitability of 16HBE as cell model for our functional studies. Using the CRISPR-Cas9 system, we created a deletion of one base pair in the AHRR DNA, disenabling the formation of functional AHRR protein by creating an early stop codon for mRNA translation in 16HBE cells [25]. CRISPR-Cas9 knockout of AHRR was confirmed at the DNA level using Sanger-sequencing. The chromatograms of the DNA sequence of WT and AHRR KO cells showed a deletion of one guanine (G) in the AHRR KO cells (Figure 3B), resulting in an early stop codon at the 75th amino acid instead of the 715th amino acid (Figure 3C). Insertion/deletion (Indel) spectrum analysis of the Sanger sequencing result showed that 88.5% of sequences had this deletion of 1 base pair (Figure 3D). As shown in Figure 3E, there were no differences in expression of AHR between WT and AHRR KO cells at baseline. Upon exposure to CSE, the expression of AHR was only significantly induced in the AHRR KO cells, with the CSE-induced increase in AHR expression being significantly stronger in the AHRR KO cells compared to WT cells (Figure 3F), reflecting the loss of regulatory control by AHRR. 

### 3.4. AHRR Knockout Attenuates the Proliferation of 16HBE Cells

**Figure 4:**
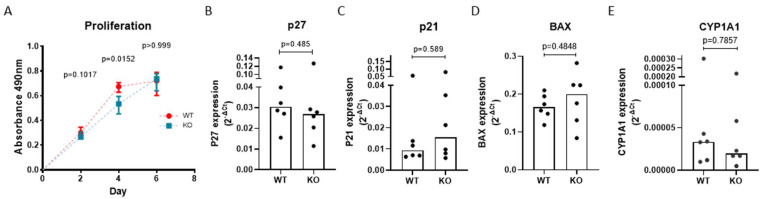
AHRR knockout attenuates the proliferation of 16HBE cells without altering AHR-related genes. 16HBE wild-type (WT) or AHRR knockout (KO) cells (*n* = 6) were seeded in triplicate in 96-wells plates, and the cell proliferation was measured using MTS assay at day 2, day 4 and day 6 after seeding. Cell proliferation rate of WT and AHRR KO cells is shown and depicted as median with interquartile range (**A**). 16HBE WT and AHRR KO cells (*n* = 6) were seeded in duplicate in 24-well plates, grown to confluence, serum-deprived overnight, incubated in medium with/without 10% CSE for 6 h and harvested for isolation of RNA. The mRNA levels of *P27* (**B**), *P21* (**C**), *BAX* (**D**) and *CYP1A1* (**E**) were determined using qPCR, related to the expression of housekeeping genes *B2M* and *PPIA* and expressed as 2^−ΔΔCt^. Median levels are indicated. The differences between WT and AHRR KO were tested using the Mann–Whitney test. *p*-values are as indicated between the groups.

As AHRR suppresses the activation of AHR and its downstream targets, its knockout was expected to increase the expression of cell cycle inhibitors p27 and p21, thereby reducing cell proliferation [28,29]. To test the effect on cell proliferation we used the MTS assay and observed that AHRR KO cells proliferate significantly slower compared to WT 16HBE cells from day 2–4 (Figure 4A). The difference between 16HBE WT and AHRR KO cells no longer existed at day 6, when 16HBE WT cells reached confluence and stopped proliferating, while AHRR KO cells were still proliferating, reflecting a lower proliferation rate. In addition to cell cycle inhibitors, AHR is known to regulate cellular apoptosis and metabolism through the expression of the apoptotic marker *BAX* and xenobiotic metabolic enzyme coding gene *CYP1A1*, which may also contribute to the reduced proliferation in AHRR KO cells. Thus, next to the mRNA expression of *P27* and *P21*, we assessed *BAX* and *CYP1A1* expression. As shown in Figure 4B–E, we did not find significant differences in the expression of these genes between 16HBE WT and AHRR KO cells at baseline. 

### 3.5. AHRR Knockout Decreases Mitochondrial Membrane Potential upon CSE Exposure 

**Figure 5:**
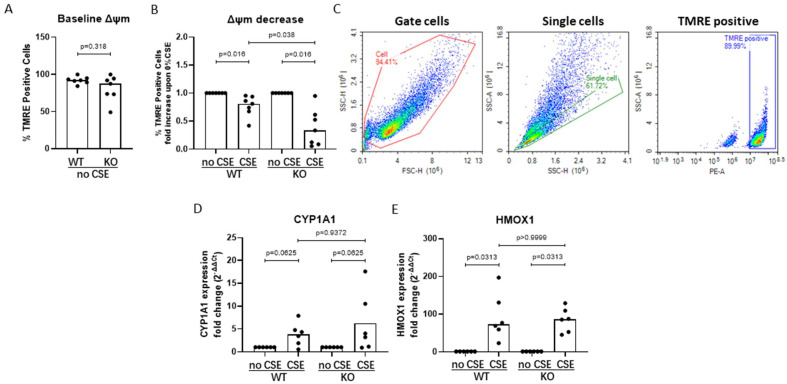
AHRR knockout enhances CSE-induced mitochondrial damage in 16HBE cells. 16HBE wild-type (WT) and AHRR knockout (KO) cells were seeded in duplicate, grown to confluence, serum-deprived overnight and incubated in medium with/without 20% CSE for 4 h before staining with TMRE and mitochondrial membrane potential (Δψm) was measured using flow cytometry (*n* = 7). For the isolation of RNA, the cells were incubated with medium control or 10% CSE for 6 h before harvesting (*n* = 6). Baseline levels of Δψm in 16HBE WT and AHRR KO cells as measured by TMRE assay (**A**). Fold change in Δψm levels upon CSE exposure compared to unexposed cells in WT and AHRR KO, respectively (**B**). Gating strategy of Δψm measurement using flow cytometry of a representative experiment is shown (**C**). As demonstrated in the left panel, live cells were selected based on cell size (forward and side scatter); as indicated in the middle panel, single cells were selected on basis of similar size on the forward and side scatter; as indicated in the right panel, TMRE positive cells were gated (intensity above 10^7^ in the PE channel). mRNA levels of *CYP1A1* (**D**) and *HMOX1* (**E**) were measured using qPCR, related to the expression of housekeeping genes *B2M* and *PPIA* and expressed as 2^−^^ΔΔCt^. Median levels are indicated. CSE groups were normalized to their medium controls. Differences between WT and AHRR KO were tested with the Mann–Whitney test, and differences between medium and CSE were tested with Wilcoxon rank-sum test. *p*-values are as indicated between the groups.

Another way in which AHRR may regulate cellular metabolic activity is through suppression of the effect of AHR on mitochondrial function, and we therefore assessed mitochondrial membrane potential. At baseline, we did not detect differences between WT and AHRR KO cells (Figure 5A). When inducing mitochondrial dysfunction by exposing the cells to CSE, however, we observed a significantly stronger reduction in membrane potential in AHRR KO cells compared to WT cells, indicating loss of mitochondrial membrane integrity (Figure 5B,C). 

To study whether the decrease in mitochondrial membrane potential was accompanied by CSE-induced oxidative stress, we assessed the expression of *CYP1A1,* a key enzyme in xenobiotic metabolism and regulator of oxidative stress, as well as oxidative stress marker *HMOX1*. At baseline, these genes were not differently expressed between WT and AHRR KO cells (online Supplemental Figure S2). Upon CSE exposure, we found a trend towards increased *CYP1A1* expression (Figure 5D) and a strong and significant increase in the expression of *HMOX1* in both WT and AHRR KO cells, without a significant difference between WT and AHRR KO cells (Figure 5E).

### 3.6. AHRR Knockout Decreases Bronchial Epithelial Cell Viability and Increases Apoptotic Cell Death upon CSE Exposure

**Figure 6:**
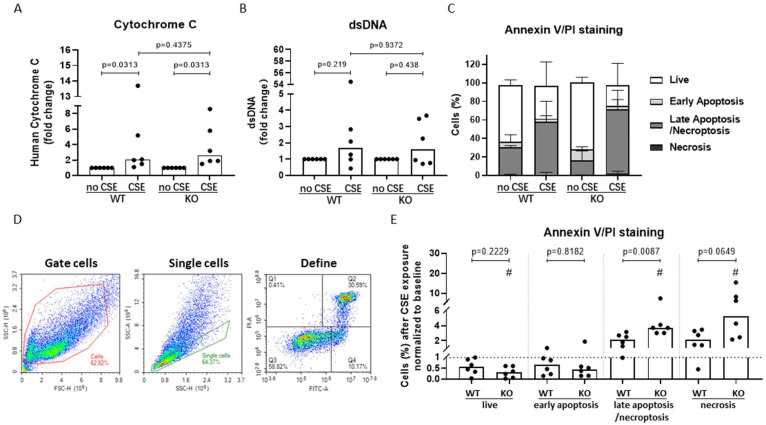
AHRR knockout enhances CSE-induced cell apoptosis/necroptosis without altering cytochrome C and dsDNA levels. 16HBE wild-type (WT) and AHRR knockout (KO) cells (*n* = 6/group) were seeded in duplicate, grown to confluence and serum-deprived overnight. For the measurement of cytochrome C and dsDNA levels, the cells were either incubated in medium with/without 20% CSE for 24 h and cell-free supernatant was collected. For the Annexin V/PI measurement, the cells were incubated in medium with/without 20% CSE for 4 h before Annexin V/PI staining was performed and measured using flow cytometry. Cytochrome C (**A**) and dsDNA (**B**) in cell-free supernatants were measured and CSE groups were normalized to their medium controls. A stack histogram of the percentages of live, early apoptotic, late apoptotic/necroptotic and necrotic cells in the single cell population (medians with upper interquartile range) is shown (**C**). Gating strategy of Annexin V/PI staining measurement using flow cytometry of a representative experiment is shown (**D**). As demonstrated in the left panel, live cells were selected based on cell size (forward and side scatter); as indicated in the middle panel, single cells were selected on basis of similar size on the forward and side scatter; and as indicated in the right panel, single cells were divided into 4 quartiles based on the positivity of Annexin V (FITC channel) and PI (PI channel). Necrosis (Q1), PI positive and Annexin V negative; late apoptosis/necroptosis (Q2), positive for both Annexin V and PI; live cells (Q3), negative for both Annexin V and PI; early apoptotic cells (Q4), Annexin V positive and PI negative. Annexin V/PI staining assay is shown as the fold change in percentage of cells upon 20% CSE compared to their respective medium control groups, indicated by the dotted line (**E**). # Indicates a significant difference in the percentage of cells upon 20% CSE exposure compared to medium control groups (no CSE), *p* < 0.05. The other *p*-values are as indicated between the WT and KO groups. Median levels are indicated. Differences between CSE and medium were assessed by Wilcoxon rank-sum test and differences between WT and AHRR KO were assessed by Mann–Whitney test.

To assess whether the loss of mitochondrial integrity was accompanied by cytochrome C release and subsequent induction of apoptotic cell death [30], we assessed the effect of AHRR knockout on CSE-induced release of cytochrome C. At the same time, double stranded DNA (dsDNA) [8] was measured as marker of necrotic cell death. At baseline, we did not find differences in both markers between WT and AHRR KO cells (online Supplemental Figure S3). Upon exposure to 20% CSE, we found a significant increase in the levels of cytochrome C, but not dsDNA in both WT and AHRR KO cells, while knockout of AHRR did not significantly alter this effect of CSE (Figure 6A,B). 

Finally, we assessed whether AHRR knockout affected the induction of different cell death modalities after CSE exposure. In AHRR KO cells we observed a trend towards more live cells and significantly less late apoptotic/necroptotic cells at baseline compared to WT cells (online Supplemental Figure S4). We observed that 20% CSE significantly reduced the percentage of viable cells and significantly increased the percentage of late apoptotic/necroptotic as well as necrotic cells only in AHRR KO cells (Figure 6C–E), while it did not significantly affect the percentage of early apoptotic cells. Of note, CSE induced a significantly stronger increase in late apoptotic cells in AHRR KO cells compared to in WT cells (Figure 6E), which is in line with the stronger loss of mitochondrial membrane potential. Together, these results suggest that AHRR KO may favor the induction of late apoptotic/necroptotic cell death upon CSE exposure.

## 4. Discussion

In this study, we hypothesized that epithelial cells from COPD patients are more susceptible to cigarette smoke-induced hypomethylation of *AHRR*, subsequently causing increased expression of *AHRR* and reduced expression of AHR, attenuating its protective effects upon cigarette smoke exposure. We observed that DNA methylation of *AHRR* at cg05575921 and cg21161138 is lower in subjects with COPD than control subjects with normal lung function, but only in active smokers, suggesting that smoking induces a stronger decrease in DNA methylation of *AHRR* in COPD. Accordingly, in airway epithelial cultures we found lower DNA methylation in COPD compared to control-derived cells at both CpG-sites, but only upon exposure to CSE. Lower levels of *AHRR* DNA methylation at cg21161138 were accompanied by a trend towards higher expression of *AHRR* mRNA. In turn, higher expression of *AHRR* correlated with lower expression levels of *AHR*. In the human bronchial cell line 16HBE, we observed that knockout of *AHRR* resulted in a lower cell proliferation rate, indicating that *AHRR* act to promote epithelial proliferation. In addition, we found that loss of *AHRR* leads to a stronger CSE-induced loss of mitochondrial integrity. This was accompanied by a stronger CSE-induced increase in programmed late apoptotic/necroptotic cell death, and *AHRR* may thus act to promote a switch to unprogrammed/necrotic cell death. Overall, our results suggest that the negative association between *AHRR* DNA methylation and COPD observed in blood is also present in airway epithelial cells, where it affects cell proliferation, mitochondrial membrane potential and the regulation of apoptotic/necroptotic cell death.

Our findings are in line with previous results of a large number of EWA studies in blood showing lower DNA methylation levels of *AHRR* upon exposure to cigarette smoke. Accordingly, our group has validated the hypomethylation levels of cg05575921 and cg21161138 of *AHRR* in lung tissue of ex- and current smokers compared to never smokers and showed its association with gene expression levels of *AHRR* [13]. Here, we only observed a trend between *AHRR* methylation at cg21161138 and *AHRR* mRNA expression in epithelial cells, potentially because of the relatively lower power due to drop-outs. Nevertheless, previous studies have shown that smoking-related hypomethylation of *AHRR* is correlated with *AHRR* mRNA expression in blood, lung tissue and lung-derived macrophages [13,31,32], suggesting that the gene expression of *AHRR* is regulated by DNA methylation. Additionally, we observed a negative correlation between the expression of *AHRR* and *AHR*, which indicates that *AHRR* indeed regulates the expression of *AHR* in human airway epithelium. This was confirmed by the stronger increase in CSE-induced *AHR* expression upon CRISPR/Cas9 knockout of AHRR in 16HBE cells. Unexpectedly, we did not observe a direct downregulatory effect of CSE on *AHRR* DNA methylation at the specific CpG sites. A possible explanation may be that longer or repeated exposure to cigarette smoke is required, as all the AECs donors were ex-smokers and the *AHRR* DNA methylation of AECs may already low at baseline. Our findings indicate that *AHRR* DNA methylation at cg05575921 and cg21161138 in blood is associated with COPD. Notably, we only found a correlation in current smokers but not never smokers and our results were confirmed in cultured airway epithelial cells, where we only observed lower *AHRR* DNA methylation upon in vitro exposure to CSE. This suggests that the airway epithelium from COPD patients is more susceptible to cigarette smoke-induced hypomethylation of *AHRR*. 

When translating our findings to COPD, the lower methylation of *AHRR* upon smoking is expected to lead to higher expression of AHRR and reduced expression of AHR. Given the protective effects of AHR signaling, this may lead to compromised protective effects against cigarette smoking exposure. Our data shows a slower proliferation rate of epithelial cells deficient of AHRR, indicating that AHRR positively affects epithelial proliferation. This is in line with the previously described role for the AHR pathway, given its effects on proliferation [33], cell cycle inhibitors as p27 and p21 and apoptosis. Excessive proliferation of airway epithelium has been associated with the loss of epithelial cell–cell contacts and epithelial-to-mesenchymal transition, which has been implicated in the dysregulation of epithelial repair responses and airway remodeling in chronic bronchitis and COPD as well as lung cancer [6,34,35,36]. In contrast to the previously described role of AHR signaling in apoptosis, we did not observe more but less (late) apoptotic cells upon AHRR KO in absence of CSE. One of the plausible explanations could be that AHRR KO induces a more senescent cell state, which is well-known to be accompanied by less apoptosis and proliferation. This might also be considered as a protective mechanism against tumorigenic responses [37,38]. It will be of interest to further study the role of AHRR in regulation of cellular senescence, which was outside the scope of the current study. While we did find impaired cell proliferation in AHRR KO cells, this was not accompanied by higher expression of *P21* and *P27*. One of the explanations may be that AHR activation regulates cell cycle by altering the phosphorylation instead of mRNA expression of *P21* and *P27* in airway epithelium, as described before [39]. In addition, the AHRR-AHR axis may regulate cell cycle in a different manner, for instance by altering the metabolic activity of cells through effects on mitochondrial function [33,40].

Similar to the lack of effects on *P27* and *P21*, knockout of AHRR did not affect epithelial expression of *CYP1A1*, in absence nor presence of CSE. Multiple studies have shown the induction of CYP1A1 through AHR pathway, identifying CYP1A1 as a target of AHR, its increased expression being the first step of metabolizing and detoxifying of drugs or toxin exposure [41,42]. Zajda et al. also showed that depending on the composition of polycyclic aromatic hydrocarbons (PAHs) mixtures, the activation of AHR could either induce the expression of *CYP1A1* or have no influence on it [43]. It is possible that baseline AHR activity in 16HBE cells nor the activation of AHR by PAHs present in the used extract of cigarette smoke leads to the expression of *CYP1A1*.

When assessing the effect of AHRR knockout on mitochondrial integrity, at baseline we did not observe any effects. Intriguingly, we found that upon CSE exposure, AHRR KO cells showed a significantly stronger decrease in mitochondrial membrane potential than WT cells. The CSE-induced loss of mitochondrial integrity was accompanied by the release of apoptosis marker cytochrome C and the induction of oxidative stress markers *HMXO1*, although these were not further increased upon AHRR knockout. A possible explanation for the lack of effect on *HMOX1* may be that the induction of *HMOX1* expression by CSE in WT cells was already very strong and may have reached a plateau phase, and thus AHRR knockout was not able to further increase it. 

Loss of mitochondrial integrity may be considered as a deleterious effect, leading to impaired mitochondrial function. On the other hand, loss of mitochondrial membrane potential is accompanied by increased mitochondrial membrane permeability, which allows cytochrome C, the trigger of apoptotic cell death to be released from the inner mitochondrial membrane to the cytosol and initiate cellular apoptosis [44,45]. In light of our previously observed CSE-induced switch from apoptotic to necrotic cell death [7], this may have beneficial effects. During programmed cell death like apoptosis and necroptosis, dying cells emit signals to recruit macrophages to phagocytose the dead cells before DAMPs are released. Therefore, reducing immunogenic cell death may act to prevent DAMP release and subsequent oxidative damage, inflammatory or immunogenic reactions which could contribute to the pathogenesis COPD [46,47]. We did not find different levels of cytochrome C nor of the DAMP dsDNA upon exposure to CSE in AHRR KO cells compared to WT cells. The degradation of cytochrome C once being released from mitochondria into the cytoplasm may have contributed here [48]. As for dsDNA, we did not observe a significant increase upon CSE exposure, indicating that higher concentrations of CSE may have been needed to observe an effect, as observed previously [8]. Nevertheless, AHRR KO cells showed a stronger induction of late cell apoptosis/necroptosis upon CSE exposure compared to WT cells. These findings may have implications for COPD, where elevated levels of AHRR as consequence of DNA hypomethylation upon smoking may contribute to persistent inflammation and tissue injury by disturbing the balance between apoptotic and necroptotic cell death. It will be of interest in future studies to assess the effect of AHRR knockout upon prolonged or repeated exposure to CSE, as well as higher concentrations to assess effects on DAMP release. 

A limitation of this study is that the DNA methylation levels of cg05575921 and cg21161138 were only available in the epithelial cultures from 4 to 11 donors after removing un-qualified pyrosequencing data. The limited power may be the reason that no different DNA methylation levels were observed at cg05575921, and the correlations between *AHRR* DNA methylation and its expression were not significant. The primary AECs were derived using bronchial brushing from a unique study with well-characterized COPD patients and smoking status and age-matched controls, which requires strict selection of the donors, thus we could not further increase the donor number for the current study. Another limitation is that the AHRR knockout cell model was created in the human bronchial epithelial cell line 16HBE and not in primary AECs. The main difficulty is to culture and scale up clones from primary epithelial cells after single cell sorting. Nevertheless, we have shown previously that 16HBE cells model primary AECs accurately with respect to the cigarette smoke-induced pro-inflammatory responses [49]. Thus, we considered the use of cell lines instead of primary AECs was a reasonable alternative to create a viable and stable AHRR knockout cell model for the functional study. 

## 5. Conclusions

We found lower DNA methylation levels cg05575921 and cg21161138 of *AHRR* in the blood from current smoking subjects with COPD compared to those without COPD. Accordingly, we found lower DNA methylation levels of these CpG-sites in AHRR upon exposure to CSE in COPD-derived airway epithelial cells compared to non-COPD control-derived airway epithelial cells. While the exact mechanism of how AHRR contributes to the pathogenesis of COPD requires further investigation, our results suggest that lower DNA methylation in COPD may cause induction of AHRR gene expression. This may lead to altered airway epithelial responses, causing dysregulation of mitochondria function and subsequently reducing programmed cell death upon cigarette smoke exposure. Therefore, it may be of interest to gain further insight into the downstream mechanisms of AHRR to assess the suitability of the identified effectors for future therapeutic strategies for COPD. 

## Figures and Tables

**Table 1:** Subject characteristics of Lifelines cohort.

	Never Smokers			Current Smokers		
	All	Non-COPD	COPD	All	Non-COPD	COPD
N	903	587	316	658	379	279
Male, N (%) *	508 (56.3)	364 (62)	144 (45.6)	375 (57)	224 (59.1)	151 (54.1)
Age (years) *^,#^ median (min-max)	46 (18–80)	44 (18–79)	50 (28–80)	46 (22–79)	44 (22–76)	49 (26–79)
Pack-years ^,#^ mean (min-max)	NA	NA	NA	19 (5.05–100)	16.4 (5.05–68.1)	22.2 (5.25–100)
FEV_1_/FVC (%) *^,#^ mean (SD)	74.5 (8.22)	79.5 (4.69)	65.2 (4.38)	71.7 (8.77)	77.9 (4.75)	63.3 (5.39)
FEV_1_ (%pred) *^#^ mean (SD)	101 (14.5)	105 (12.6)	92.6 (14.3)	94.4 (14.9)	101 (12)	85.8 (14)

FEV_1_: forced expiratory volume in 1 s; FVC: forced vital capacity. * Significant differences (*p* < 0.05) between non-COPD and COPD subjects in never smokers (Χ^2^ test was applied to categorical data. Mann–Whitney U test or unpaired *t*-test was applied to non-normal distributed and normal distributed quantitative data, respectively). # Significant differences (*p* < 0.05) between non-COPD and COPD in current smokers (Χ^2^ test was applied to categorical data. Mann–Whitney U test or unpaired *t*-test were applied to non-normal distributed and normal distributed quantitative data, respectively).

**Table 2:** Subject characteristics.

Subject	Non-COPD Control (*n* = 8)	COPD (*n* = 14)
Age (years), median (min-max)	61 (52–63)	56.5 (39–61)
Male (n/all)	5/8	6/14
Pack years, median (min-max)	33 (17–64)	39 (6–64)
% Predicted FEV1, median (min-max) *	111.63 (102.46–123.95)	47.69 (16.58–78.4)

Data are presented as n/total or median (range). FEV1: forced expiratory volume in 1 s. FEV_1_% predicted: percentage of predicted FEV_1_. * Significant differences (*p* < 0.05) between non-COPD controls and COPD patients (Χ^2^ test was applied to categorical data and Mann–Whitney U test to quantitative data, respectively).

**Table 3:** Primers used for DNA methylation analysis.

Primer Name	Sequence (5′ to 3′)
Amplification primers	
FW_AHRR_cg05575921	GGGGATTGTTTATTTTTGAGAGGGTAG
RV_AHRR_cg05575921	ACCTATCCCCTACCTCCC
FW_AHRR_cg21161138	ATTTTGTAGGGGTTTTGGTGGT
RV_AHRR_cg21161138	CAACTCTAACCCCAAAATCTCT
Sequencing primers	
SEQ_AHRR_cg05575921	ATTTTTGAGAGGGTAGT
SEQ_AHRR_cg21161138	GGGTTTTGGTGGTTG

**Table 4:** Taqman probes.

Gene Name	Cat. Number of Taqman Probs
*B2M*	Hs99999907_m1
*PPIA*	Hs99999904_m1
*AHRR*	Hs01005075_m1
*AHR*	Hs00169233_m1
*P27*	Hs00153277_m1
*P21*	Hs00355782_m1
*BAX*	Hs00180269_m1
*CYP1A1*	Hs01054796_g1
*HMOX1*	Hs00157965_m1

## Data Availability

Summary statistics of the analysis are available upon reasonable request by contacting the corresponding author.

## Supplementary Materials

The following supporting information can be downloaded at: https://www.mdpi.com/article/10.3390/cells11213423/s1, Figure S1. CSE exposure does not affect DNA methylation of AHRR in airway epithelial cells (AECs); Figure S2. CYP1A1 and HMOX1 expression in 16HBE cells does not change upon AHRR knockout; Figure S3. Release of cytochrome C and dsDNA in 16HBE cells does not change upon AHRR knockout (KO); Figure S4. Cell death modalities in 16HBE wild-type (WT) and AHRR knockout (KO) cells measured by Annexin V/PI staining.

## References

[1] (2021). Global Strategy for the Diangosis, Management, and Prevention of Chronic Obstructive Pulmonary Disease 2021 Report.

[2] Barnes P.J., Burney P.G.J., Silverman E.K., Celli B.R., Vestbo J., Wedzicha J.A., Wouters E.F.M. (2015). Chronic obstructive pulmonary disease. Nat. Rev. Dis. Prim..

[3] Chen Q., de Vries M., Nwozor K.O., Noordhoek J.A., Brandsma C.-A., Boezen H.M., Heijink I.H. (2021). A Protective Role of FAM13A in Human Airway Epithelial Cells Upon Exposure to Cigarette Smoke Extract. Front. Physiol..

[4] Hiromichi H., Jun A., Saburo I., Kenji K., Naoki T., Yutaka Y., Hiroshi W., Jun K., Kenichiro S., Takanori N. (2013). Mitochondrial fragmentation in cigarette smoke-induced bronchial epithelial cell senescence. Am. J. Physiol. Lung Cell. Mol. Physiol..

[5] Hoffmann R.F., Zarrintan S., Brandenburg S.M., Kol A., de Bruin H.G., Jafari S., Dijk F., Kalicharan D., Kelders M., Gosker H.R. (2013). Prolonged cigarette smoke exposure alters mitochondrial structure and function in airway epithelial cells. Respir. Res..

[6] Aghapour M., Raee P., Moghaddam S.J., Hiemstra P.S., Heijink I.H. (2018). Airway epithelial barrier dysfunction in chronic obstructive pulmonary disease: Role of cigarette smoke exposure. Am. J. Respir. Cell Mol. Biol..

[7] Van Der Toorn M., Slebos D.-J., De Bruin H.G., Leuvenink H.G., Bakker S.J.L., Gans R.O.B., Koëter G.H., Van Oosterhout A.J.M., Kauffman H.F. (2007). Cigarette smoke-induced blockade of the mitochondrial respiratory chain switches lung epithelial cell apoptosis into necrosis. Am. J. Physiol. Lung Cell Mol. Physiol..

[8] Pouwels S.D., Zijlstra G.J., van der Toorn M., Hesse L., Gras R., Ten Hacken N.H.T., Krysko D.V., Vandenabeele P., De Vries M., Van Oosterhout A.J.M.M. (2016). Cigarette smoke-induced necroptosis and DAMP release trigger neutrophilic airway inflammation in mice. Am. J. Physiol. Cell Physiol. Lung Cell. Mol. Physiol..

[9] Pouwels S.D., Heijink I.H., Ten Hacken N.H.T., Vandenabeele P., Krysko D.V., Nawijn M.C., Van Oosterhout A.J.M. (2014). DAMPs activating innate and adaptive immune responses in COPD. Mucosal Immunol..

[10] Janciauskiene S. (2020). The beneficial effects of antioxidants in health and diseases. Chronic Obstr. Pulm. Dis..

[11] Nyunoya T., Mebratu Y., Contreras A., Delgado M., Chand H.S., Tesfaigzi Y. (2014). Molecular processes that drive cigarette smoke-induced epithelial cell fate of the lung. Am. J. Respir. Cell Mol. Biol..

[12] Moore L.D., Le T., Fan G. (2013). DNA methylation and its basic function. Neuropsychopharmacology.

[13] De Vries M., Van Der Plaat D.A., Nedeljkovic I., Verkaik-Schakel R.N., Kooistra W., Amin N., Van Duijn C.M., Brandsma C.A., Van Diemen C.C., Vonk J.M. (2018). From blood to lung tissue: Effect of cigarette smoke on DNA methylation and lung function. Respir. Res..

[14] Bojesen S.E., Timpson N., Relton C., Davey Smith G., Nordestgaard B.G. (2017). AHRR (cg05575921) hypomethylation marks smoking behaviour, morbidity and mortality. Thorax.

[15] Kodal J.B., Kobylecki C.J., Vedel-Krogh S., Nordestgaard B.G.G., Bojesen S.E. (2018). AHRR hypomethylation, lung function, lung function decline and respiratory symptoms. Eur. Respir. J..

[16] Mimura J., Fujii-Kuriyama Y. (2003). Functional role of AhR in the expression of toxic effects by TCDD. Biochim. Biophys. Acta.

[17] Yamaguchi M., Hankinson O. (2020). An aryl hydrocarbon receptor agonist suppresses the growth of human umbilical vein endothelial cells in vitro: Potent effect with polyunsaturated fatty acids. Int. J. Exp. Pathol..

[18] Formosa R., Borg J., Vassallo J. (2017). Aryl hydrocarbon receptor (AHR) is a potential tumour suppressor in pituitary adenomas. Endocr. Relat. Cancer.

[19] Guerrina N., Traboulsi H., Eidelman D.H., Baglole C.J. (2018). The Aryl Hydrocarbon Receptor and the Maintenance of Lung Health. Int. J. Mol. Sci..

[20] Nothdurft S., Thumser-Henner C., Breitenbücher F., Okimoto R.A., Dorsch M., Opitz C.A., Sadik A., Esser C., Hölzel M., Asthana S. (2020). Functional screening identifies aryl hydrocarbon receptor as suppressor of lung cancer metastasis. Oncogenesis.

[21] Haarmann-Stemmann T., Abel J. (2006). The arylhydrocarbon receptor repressor (AhRR): Structure, expression, and function. Biol. Chem..

[22] Sijtsma A., Rienks J., van der Harst P., Navis G., Rosmalen J.G.M., Dotinga A. (2022). Cohort Profile Update: Lifelines, a three-generation cohort study and biobank. Int. J. Epidemiol..

[23] De Vries M., Van Der Plaat D.A., Vonk J.M., Boezen H.M. (2018). No association between DNA methylation and COPD in never and current smokers. BMJ Open Respir. Res..

[24] Heijink I.H., De Bruin H.G., Dennebos R., Jonker M.R., Noordhoek J.A., Brandsma C.A., Van Den Berge M., Postma D.S. (2016). Cigarette smoke-induced epithelial expression of WNT-5B: Implications for COPD. Eur. Respir. J..

[25] Broome S.T., Fisher T., Faiz A., Keay K.A., Musumeci G., Al-Badri G., Castorina A. (2021). Assessing the anti-inflammatory activity of the anxiolytic drug buspirone using crispr-cas9 gene editing in lps-stimulated bv-2 microglial cells. Cells.

[26] Pouwels S.D., Wiersma V.R., Fokkema I.E., Berg M., ten Hacken N.H.T., van den Berge M., Heijink I., Faiz A. (2021). Acute cigarette smoke-induced eQTL affects formyl peptide receptor expression and lung function. Respirology.

[27] Hahn M.E., Allan L.L., Sherr D.H. (2009). Regulation of Constitutive and Inducible AHR Signaling: Complex Interactions Involving the AHR Repressor. Biochem. Pharmacol..

[28] Liu Y., Liang X., Yin X., Lv J., Tang K., Ma J., Ji T., Zhang H., Dong W., Jin X. (2017). Blockade of IDO-kynurenine-AhR metabolic circuitry abrogates IFN-γ-induced immunologic dormancy of tumor-repopulating cells. Nat. Commun..

[29] Koliopanos A., Kleeff J., Xiao Y., Safe S., Zimmermann A., Büchler M.W., Friess H. (2002). Increased arylhydrocarbon receptor expression offers a potential therapeutic target for pancreatic cancer. Oncogene.

[30] Kalpage H.A., Bazylianska V., Recanati M.A., Fite A., Liu J., Wan J., Mantena N., Malek M.H., Podgorski I., Heath E.I. (2019). Tissue-specific regulation of cytochrome c by post-translational modifications: Respiration, the mitochondrial membrane potential, ROS, and apoptosis. FASEB J..

[31] Takeuchi F., Takano K., Yamamoto M., Isono M., Miyake W., Mori K., Hara H., Hiroi Y., Kato N. (2022). Clinical Implication of Smoking-Related Aryl-Hydrocarbon Receptor Repressor (AHRR) Hypomethylation in Japanese Adults. Circ. J..

[32] Monick M.M., Beach S.R.H., Plume J., Sears R., Gerrard M., Brody G.H., Philibert R.A. (2012). Coordinated changes in AHRR methylation in lymphoblasts and pulmonary macrophages from smokers. Am. J. Med. Genet. B Neuropsychiatr. Genet..

[33] Jia Y., Zhao Y., Zhang Z., Shi L., Fang Y., Chang C. (2021). Aryl hydrocarbon receptor signaling pathway plays important roles in the proliferative and metabolic properties of bone marrow mesenchymal stromal cells. Acta Biochim. Biophys. Sin..

[34] Milara J., Peiró T., Serrano A., Cortijo J. (2013). Epithelial to mesenchymal transition is increased in patients with COPD and induced by cigarette smoke. Thorax.

[35] Barnes P.J., Shapiro S.D., Pauwels R.A. (2003). Chronic obstructive pulmonary disease: Molecular and cellular mechanisms. Eur. Respir. J..

[36] Hackett T.L., Warner S.M., Stefanowicz D., Shaheen F., Pechkovsky D.V., Murray L.A., Argentieri R., Kicic A., Stick S.M., Bai T.R. (2009). Induction of epithelial-mesenchymal transition in primary airway epithelial cells from patients with asthma by transforming growth factor-β1. Am. J. Respir. Crit. Care Med..

[37] Kondrikov D., Elmansi A., Bragg R.T., Mobley T., Barrett T., Eisa N., Kondrikova G., Schoeinlein P., Aguilar-Perez A., Shi X.M. (2020). Kynurenine inhibits autophagy and promotes senescence in aged bone marrow mesenchymal stem cells through the aryl hydrocarbon receptor pathway. Exp. Gerontol..

[38] Calcinotto A., Kohli J., Zagato E., Pellegrini L., Demaria M., Alimonti A. (2019). Cellular Senescence: Aging, Cancer, and Injury. Physiol. Rev..

[39] Hsu Y.H., Chang C.C., Yang N.J., Lee Y.H., Juan S.H. (2014). RhoA-mediated inhibition of vascular endothelial cell mobility: Positive feedback through reduced cytosolic p21 and p27. J. Cell. Physiol..

[40] Ren R., Fang Y., Sherchan P., Lu Q., Lenahan C., Zhang J.H., Zhang J., Tang J. (2022). Kynurenine/Aryl Hydrocarbon Receptor Modulates Mitochondria-Mediated Oxidative Stress and Neuronal Apoptosis in Experimental Intracerebral Hemorrhage. Antioxid. Redox Signal..

[41] Lamas B., Natividad J.M., Sokol H. (2018). Aryl hydrocarbon receptor and intestinal immunity. Mucosal Immunol..

[42] Yuan J., Sun X., Che S., Zhang L., Ruan Z., Li X., Yang J. (2021). AhR-mediated CYP1A1 and ROS overexpression are involved in hepatotoxicity of decabromodiphenyl ether (BDE-209). Toxicol. Lett..

[43] Zajda K., Ptak A., Rak A., Fiedor E., Grochowalski A., Milewicz T., Gregoraszczuk E.L. (2017). Effects of human blood levels of two PAH mixtures on the AHR signalling activation pathway and CYP1A1 and COMT target genes in granulosa non-tumor and granulosa tumor cell lines. Toxicology.

[44] Li Y., Zhou Y., Wang M., Lin X., Zhang Y., Laurent I., Zhong Y., Li J. (2021). Ampelopsin Inhibits Breast Cancer Cell Growth through Mitochondrial Apoptosis Pathway. Biol. Pharm. Bull..

[45] Lin C., Guo Y., Xia Y., Li C., Xu X., Qi T., Zhang F., Fan M., Hu G., Zhao H. (2021). FNDC5/Irisin attenuates diabetic cardiomyopathy in a type 2 diabetes mouse model by activation of integrin αV/β5-AKT signaling and reduction of oxidative/nitrosative stress. J. Mol. Cell. Cardiol..

[46] Vandenabeele P., Galluzzi L., Vanden Berghe T., Kroemer G. (2010). Molecular mechanisms of necroptosis: An ordered cellular explosion. Nat. Rev. Mol. Cell Biol..

[47] Mizumura K., Cloonan S.M., Nakahira K., Bhashyam A.R., Cervo M., Kitada T., Glass K., Owen C.A., Mahmood A., Washko G.R. (2014). Mitophagy-dependent necroptosis contributes to the pathogenesis of COPD. J. Clin. Invest..

[48] Ferraro E., Pulicati A., Cencioni M.T., Cozzolino M., Navoni F., Di Martino S., Nardacci R., Carrì M.T., Cecconi F. (2008). Apoptosome-deficient Cells Lose Cytochrome c through Proteasomal Degradation but Survive by Autophagy-dependent Glycolysis. Mol. Biol. Cell.

[49] Heijink I.H., De Bruin H.G., Van Den Berge M., Bennink L.J.C., Brandenburg S.M., Gosens R., Van Oosterhout A.J., Postma D.S. (2013). Role of aberrant WNT signalling in the airway epithelial response to cigarette smoke in chronic obstructive pulmonary disease. Thorax.

