# Supplementary material for: From Differential DNA Methylation in COPD to Mitochondria: Regulation of AHRR Expression Affects Airway Epithelial Response to Cigarette Smoke
Source: Cells. 2022 Oct 29;11(21):3423. doi: 10.3390/cells11213423 (PMC9656229; doi:10.3390/cells11213423)
Supplement: Supplementary file 1 [file cells-11-03423-s001.zip › cells-1879181-supplementary.pdf]

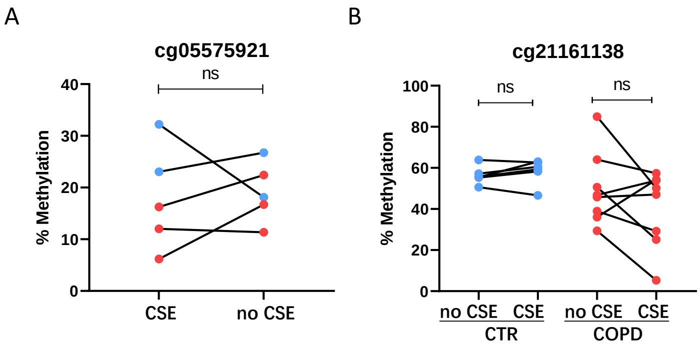

Online Supplemental Figure S1. CSE exposure does not affect DNA methylation of *AHRR* in airway epithelial cells (AECs). AECs from 6 ex-smoking non-COPD controls (CTR) and 8 COPD (GOLD stage II-IV) patients were cultured until confluence, hormonally-deprived overnight and incubated in medium with or without 7.5% CSE for 6 hours. DNA was isolated for assessment of DNA methylation and measured at the CpG-sites cg05575921 **(A)** and cg21161138 **(B)** of *AHRR* by pyrosequencing. Samples that did not pass the pyrosequencing quality control were excluded. In panel A, non-COPD controls (CTR) and COPD patients were not separated for cg05575921 due to small numbers of donors available for this CpG-site. Blue dots, non-COPD controls; red dots, COPD patients. The difference between medium control (no CSE) and CSE groups was tested using Wilcoxon signed-rank test and a p-value <0.05 was considered statistically significant.

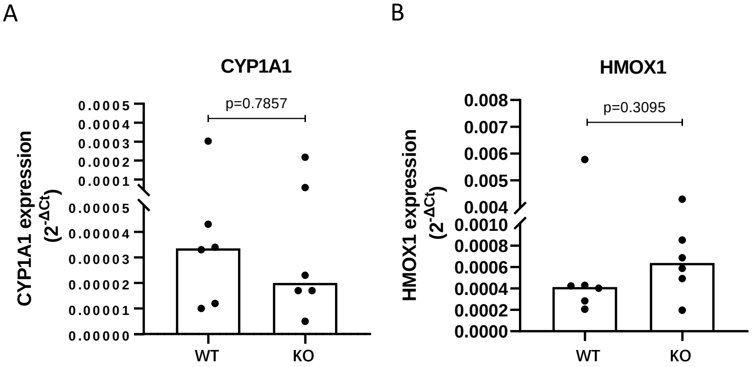

Online Supplemental Figure S2. ***CYP1A1* and *HMOX1* expression in 16HBE cells does not change upon AHRR knockout. (KO)** 16HBE wild-type (WT) and AHRR KO cells were seeded in duplicate in 24-wells plates (n=6). When 90% confluence was reached, cells were serum-deprived, incubated in medium for another 6 hours, and harvested for isolation of RNA. The mRNA expression levels of *CYP1A1* **(A)** and *HMOX1* **(B)** were determined using qPCR, related to the expression of housekeeping genes *B2M* and *PPIA* and expressed as $2^{-\Delta Ct}$. Median levels are indicated. The differences between WT and AHRR KO was tested with the Mann-Whitney test. P-values are as indicated between the WT and AHRR KO groups.

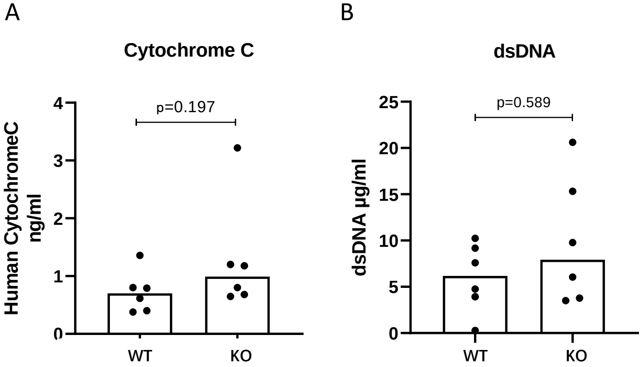

Online Supplemental Figure S3. Release of cytochrome C and dsDNA in 16HBE cells does not change upon AHRR knockout (KO). 16HBE wild-type (WT) and AHRR KO cells were seeded in duplicate in 24-wells plates (n=6). When 90% confluence was reached, cells were serum-deprived, incubated in medium for another 24 hours and cell-free supernatant was collected. Cytochrome C **(A)** and dsDNA **(B)** was measured in cell-free supernatants. Median levels are indicated. The difference between WT and AHRR KO was tested with the Mann-Whitney test. P-values are as indicated between the groups.

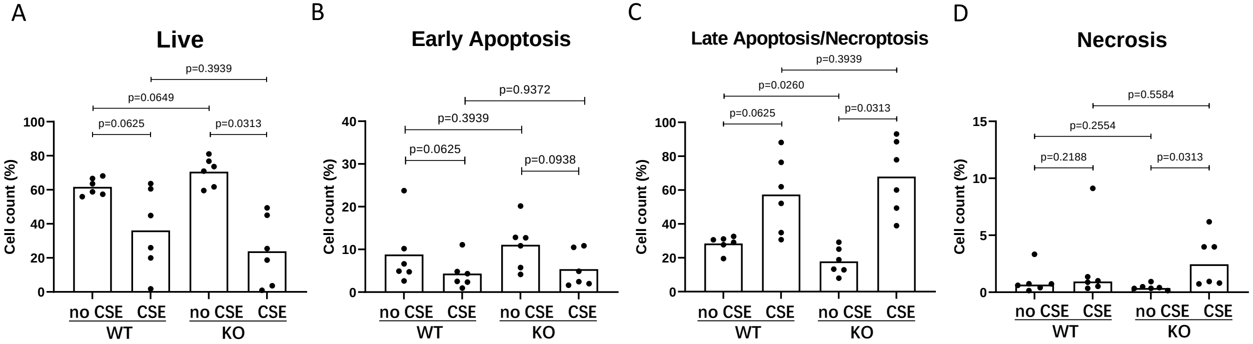

Online Supplemental Figure S4. Cell death modalities in 16HBE wild-type (WT) and AHRR knockout (KO) cells measured by Annexin V/PI staining. 16HBE WT and AHRR KO cells were seeded in duplicate in 24-wells plates (n=6). When 90% confluence was reached, the cells were serum-deprived and incubated in medium with/without 20% CSE for 4 hours before Annexin V/PI staining was performed and measured using flow cytometry. Cell counts are shown as percentage of live cells **(A)**, early apoptosis cells **(B)**, late apoptosis/necroptosis **(C)** and necrosis **(D)** are shown and median levels are depicted. The differences between WT and AHRR KO were tested with the Mann-Whitney test. P-values are as indicated between the groups.
